# Where news could not inspire change: TRT World as a party broadcaster

**DOI:** 10.1177/14648849211033444

**Published:** 2021-07-16

**Authors:** Mona Elswah, Philip N. Howard

**Affiliations:** University of Oxford, UK

**Keywords:** AKP, authoritarian regimes, international broadcasting, media freedom, propaganda, public diplomacy, TRT World, Turkey

## Abstract

Turkey has vastly increased the scale of its investment in public diplomacy tools. Although Turkey is considered one of the world’s worst jailers of journalists, its media market is one of the fastest-growing in the world. In 2015, the Istanbul-based English-language TRT World was launched with the slogan ‘where news inspires change’, The channel promised to provide impartial coverage of global news, with its experienced journalists addressing global audiences. In this study, we investigate the interplay between public diplomacy and editorial policies at TRT World. After conducting in-depth interviews with TRT World journalists, we argue that the channel has shifted its style from being Turkey’s public diplomacy tool into becoming the AKP’s voice to the world. By examining TRT World, this study provides a framework to understand how international broadcasters operate in countries where media freedom is restricted.

There is a pressing question about the conditions under which international broadcasters could succeed in their role as public diplomacy tools. This question becomes even more significant when an international broadcaster originates in a country considered one of the world’s worst jailers of journalists (Committee to Protect Journalists, 2019). Turkey has overseen a dramatic deterioration in media freedom, particularly after the failed coup attempt in 2016. Reflecting this heavy media crackdown, Turkey was ranked 153 out of 180 in the Press Freedom Index in 2021 (Reporters Without Borders, 2021). The Turkish government’s punitive measures against media have included seizing control over many outlets, closing several others and arresting dozens of journalists (Freedom House, 2019).

Despite that, the Turkish media market is one of the fastest-growing globally, with a market value of $11.6 billion and an annual growth of 11.4 percent (Yesil, 2016). When Recep Tayyip Erdoğan’s Justice and Development Party, known as AKP (the Turkish acronym), came into power in 2002, a lot of funding was allocated for Turkey’s public diplomacy activities, including international broadcasting. In 2015, the world was introduced to the Istanbul-based English-language TRT World, which promoted itself with the slogan ‘where news inspires change’. The channel promised to provide impartial coverage of global news with experienced journalists to promote Turkey’s image to overseas audiences. In a few years, TRT World expanded to include other international bureaus, with many foreign offices across the world. TRT World is one of the latest additions to the TRT Corporation (Turkish Radio and Television Corporation), which advertises itself as the Turkish national public broadcaster.

Although the Turkish media market has been well-examined over the years, Turkey’s only English-language international broadcaster remains unexplored. By interviewing former and current journalists at TRT World, this study is the first to analyse the editorial process in TRT World and its influence on the channel’s public diplomacy role. We argue that TRT World’s model of journalism was impacted by the censored and controlled media ecosystem in Turkey. Although the launch of this channel represented a fresh and ambitious change that presented an opportunity to alter the way Turkish media is perceived by global audiences, TRT World is now another reflection of how local Turkish media operates. The channel is now a by-product of the Turkish President Erdoğan’s enduring repression of the media. Here, we present three major reasons that could explain why TRT World has wandered off the track. First, its organisational model sought to recognise the Turkification of the channel since its inception in 2015. Second, the embedded censorship in the structure of the decision-making process in relation to stories concerning the Middle East. Third, the ambiguity of the regional and international role of the channel.

We conclude that TRT World has transformed its style from being Turkey’s public diplomacy tool into becoming the AKP’s voice to the world. By analysing TRT World, we provide a framework to understand how international broadcasters operate in countries where media freedom is limited. This study advances the scholarship on the relationship between public diplomacy, international broadcasting and authoritarianism.

## International broadcasting and public diplomacy

Although public diplomacy has been practiced throughout history, the term itself is relatively recent. The concept was first coined in 1965 by Edmund Gullion when he realised the need to use media in diplomatic practices (Cull, 2013). Public diplomacy envisages communicating directly with other foreign publics with the aim of ultimately influencing their governments (Malone, 1985). Public diplomacy is an official policy employed by governments to translate soft power resources into action (Gilboa, 2008; Nye, 2008). In general, there are five components of public diplomacy: listening, advocacy, cultural diplomacy, exchange diplomacy and international broadcasting (Cull, 2008). International broadcasting plays a vital role in attracting global audiences and promoting positive messages and images of a country (Youmans and Powers, 2012).

International broadcasting – through radio, television or web-based broadcasting – has changed the way public diplomacy operates (Youmans and Powers, 2012). International broadcasters are state-backed news and entertainment outlets directed at foreign audiences to shape their opinions (Price, 2003; Youmans and Powers, 2012). With the discovery of shortwaves, ideologically and politically charged state-sponsored global broadcasters emerged during World War II and the Cold War (Anderson, 2005). The BBC World Service, Deutsche Welle and the Voice of America were among the first examples of international broadcasters.

The advancement of satellite technologies has revolutionised international broadcasting. China’s CGTN (formerly CCTV), Russia’s RT (formerly Russia Today) and Iran’s Press TV emerged as examples of state-sponsored international English-language broadcasting outlets. These channels became part of the global communication landscape and challenged the dominance of Western media. They were able to generate millions of engagements on social media platforms compared with the British BBC (Bright et al., 2020).

International broadcasters have various styles and purposes. These styles include altering voices, molding opinions, destabilising nations or achieving public diplomacy goals (Price, 2003). International broadcasting styles are dependent on foreign policy goals, the domestic media landscape in a country and the extent of the political involvement in media entities (Price, 2003). The domestic socio-political factors of a country can alter and evolve the editorial structure and style of an international broadcaster (Elswah & Howard, 2020).

In the literature of international broadcasting, the term ‘public diplomacy’ is sometimes indistinguishable from ‘propaganda’, especially in relation to international broadcasting. Chang and Lin (2014) argue that the term ‘propaganda’ is more likely to be used to describe non-Western English-language international broadcasters. It is indeed challenging sometimes to find radical differences between the two terms or, to paraphrase Rawnsley (2015), one person’s public diplomacy may be another person’s propaganda. In scholarship, the distinctive element that separates the two concepts is credibility. Propaganda relies on deception, sensationalism and controlling information so the audience feels compelled to accept the message, while public diplomacy is achieved through a truthful and factual exposition of a nation’s foreign policy (Nye, 2008; Zaharna, 2004). This credibility is threatened when an international broadcaster transforms into a government mouthpiece through political interference with the newsroom.

The lack of credibility and editorial freedom can obstruct international broadcasters from achieving their public diplomacy mission (Cull, 2008). When Rawnsley (2015) examined the Russian RT, he found that RT had turned into a propaganda tool to serve the Kremlin’s agenda, providing fixed critical coverage of the US. Similarly, Al Jazeera Arabic has increasingly functioned as a propagandistic agent following the 2011 Arab Spring because of Doha’s growing control over the channel’s editorial policy (Abdul-Nabi, 2017). In comparison, Cull (2008) presented the BBC as an example of a broadcaster that was able to escape the reputation of propaganda by emphasising the credibility and impartiality of its coverage.

Scholars have examined the role of the aforementioned international broadcasters and many others in public diplomacy by analysing their messages (Dajani et al., 2019; Miazhevich, 2018; Nelson, 2019; Rawnsley, 2015), sources (Hernández and Madrid-Morales, 2020), distribution and online presence (Bright et al., 2020; Geniets, 2013; Rebello et al., 2020) and strategies and effectiveness (Orttung and Nelson, 2018). However, these channels’ internal editorial processes and styles are rarely examined. This could be explained by the challenges related to gaining access to these institutions and journalists’ self-censorship, especially in organisations backed by authoritarian governments (Elswah & Howard, 2020; Çevik, 2021; Figenschou, 2010).

## Turkish public diplomacy and international broadcasting

While it remains challenging for academics to examine these broadcasters, authoritarian governments have scaled up their media production capabilities to achieve international prominence (Noya, 2008; Walker, 2016). Turkey has certainly invested in its public diplomacy products in order to present itself as a moral country that values human rights and freedom (Sancar, 2015). It has employed persuasive public diplomacy tactics to enhance its image worldwide by promoting its popular culture, investing in international broadcasting and enhancing its rhetorical branding (Al-Ghazzi and Kraidy, 2013; Donelli, 2019). Thus, Turkey established various institutions for educational exchanges, advocacy, cultural diplomacy and media outlets to fulfill the country’s goal of becoming amongst the global powers (Çevik, 2021).

Following the coup attempt in 2016, Turkey moved from a proactive public diplomacy to a reactive one, turning its focus to combatting the negative news about the country and forming a positive public opinion about Erdoğan’s government (Çevik, 2021). In addition, the government has further utilised the use of international broadcasting to curb the negative impact of the coup attempt globally (Çevik, 2021). Today, Turkey’s English-language international broadcaster TRT World is considered an integral part of the country’s public diplomacy activities.

TRT World’s parent organisation, the Turkish Radio and Television Corporation (TRT), has long supported and promoted the plans of the Turkish government (Samborowska and Dawidczyk, 2018). Since the establishment of TRT in the 1960s as a Public Service Broadcaster (PSB), the autonomy of the corporation has been problematic (Sümer and Taş, 2020). In the early days, the first TRT Law guaranteed the corporation’s administrative autonomy and emphasised that the state would only intervene in the content related to matters of national security (Sümer and Taş, 2020). Yet, this autonomy soon diminished after the amendment to the TRT Law in 1972 that granted the government more control (Önen and Imik Tanyildizi, 2010; Sümer and Taş, 2020). The government became involved in TRT in different ways including its content production and staff recruitment (Önen and Imik Tanyildizi, 2010). This intervention prevented the corporation from effectively employing the ethos of public service broadcasting (Sümer and Adaklı, 2010).

With the AKP in power in 2002, the media structure in Turkey has transformed and reorganised drastically. TRT was restructured and controlled by people close to the AKP leaders (Inceoglu et al., 2020; Irak, 2016). Thus, TRT became strongly in line with the AKP’s messages and gave the party’s members more time on-air compared to their opponents during election times (Inceoglu et al., 2020). In 2018, the corporation became overseen by the Directorate of Communications, meaning that TRT came under the direct control of the President from that point (European Commission, 2020).

Despite that, TRT continued its expansion across the world. The corporation currently owns several channels and foreign bureaus in 27 countries, reaching 250 million people. In 2015, President Erdoğan celebrated the launch of TRT’s English-language channel ‘TRT World’ that intended to disrupt Western dominance of media (Yesil, 2020). During the launch, Erdoğan criticised the Western media coverage of Turkish stories and its ‘biased’ representation of Turkey (Yesil, 2020). In addition, TRT World runs a research centre that organises what is called the ‘TRT WORL FORUM’, for which Erdoğan tops the list of speakers (TRT World Forum, 2019).

Despite its growing significance, a scholarly examination of the TRT World’s role in public diplomacy is scarce. In this article, we take a deep dive into the backrooms of Turkey’s first English-language international broadcasters providing hard-to-achieve insights into the editorial practices of TRT World. We examine the channel’s journalistic values, the decision-making process and the evolution of its editorial policies. We ask:

- What are the factors that influence the news production process in TRT World?
- To what extent can TRT World perform as a public diplomacy tool?
- What have been the main consequences of political crises on the editorial policies of TRT World?

## Methods

To understand the intersection between public diplomacy and international broadcasting in contemporary Turkey, we carried out semi-structured in-depth interviews with 16 former and current TRT World journalists from January 2018 to March 2019. The recruitment process targeted journalists who have spent at least several months working for the channel at any of its bureaus. In some cases, journalists declined our interview requests as an act of self-censorship, for fear of losing their jobs, or because they had signed NDAs (Non-Disclosure Agreements). In spite of these challenges and by using snowball-sampling, we were able to interview a diverse group of respondents that represented the various waves and stages of development that the channel has experienced since it was established in 2015.

For consistency, the interviewing process was done by the first author, who is a Muslim Middle Eastern woman. A researcher’s race, gender, profession, religion and nationality can either constitute complicating factors or facilitate building a rapport (Feldman et al., 2004). In our case, the identity of the researcher mattered in ways we did not anticipate. This enabled us to build a rapport with the respondents and to gain their trust more effectively. We were able to discuss the Islamic orientation of the channel, the positioning of Turkey in the Middle East, the coverage of the Muslim Brotherhood and other political groups, the situation in Syria and the role of political crises in shaping the dynamics of the channel.

Although more than half of the interviews were conducted face-to-face, we had to interview the rest of the respondents, especially those based in Istanbul, via a VoIP tool. We conducted the interviews in two main locations: London and Washington DC. We decided not to conduct the face to face interviews physically in Istanbul to protect the respondents. Before conducting the interview, respondents signed written consent forms, and they were given the opportunity to ask questions about the study. The interviews ranged in duration from 40 minutes to 3 hours, with an average of 1 hour.

To ensure the validity of the interviews, we asked all the respondents the same set of questions with some minor variations. The semi-structured interview guide provided a set of questions that covered four key elements (1) the organisational structure of TRT World, (2) the channel’s editorial policies, (3) the political crises that shaped news at TRT World and (4) the editorial freedom of the channel.

In addition to writing detailed field notes, memos were produced following each interview, which were later aggregated and analysed. Through the synthesis memos, we were able to recognise the emergent themes and to conceptualise the patterns that emerged through the interviews. As we were concerned about the respondents’ safety, we used name codes to refer to them in this study. We also avoided using any quotes that could identify our respondents.

## Analysis

### Establishing a new public diplomacy tool

Drawing inspiration from the Qatari Al Jazeera, the Turkish government launched the English-language channel TRT World in 2015. This channel is part of the national broadcaster ‘The Turkish Radio and Television Corporation’ (TRT). According to one of our respondents, the other TRT-managed channels suffered budget-cuts and were resized to give the space and funds to the new TRT World channel.

All respondents agreed that the channel’s main and first goal was to transmit the Turkish point of view to the world and give voice to the Turkish culture. At first, the management intended to use the channel as a public diplomacy tool.

TRT World, the English-language channel, is trying to be that voice. It is trying to give a Turkish perspective on international news and to also reflect the Turkish perspective on domestic politics to the world. The world is growing interest in Turkish affairs at the moment, and so there is a vacuum that needs to be filled (Participant#9, 2018).

In addition to portraying Turkey positively and counterbalance the Western coverage, our respondents pointed out that TRT World intended to cover stories that fall off the radar and to give a space for marginalised communities. One of the respondents commented that TRT World would cover stories coming from countries and regions that are under-reported, such as Afghanistan, Myanmar and Africa.

In 2015, a London-based news-launching company reached out to experienced freelancers across the world to build a team to establish TRT World in Istanbul. Journalists from Al Jazeera English, Reuters, BBC and Sky News were brought in to help run the channel. Participant#12, a former TRT World journalist, stated:

TRT went to the best journalists they could reasonably get. They tried to hire credible, international reporters for TRT World, and the kinds of people you think would not put up with a completely compromised news channel (Participant#12, 2018).

Participant #12’s statement pointed out to how the management intended to establish a well-respected English-language channel that would rival the likes of Al Jazeera. This goal was communicated to all of our respondents when they were hired.

Every news organisation has a guide to house a style and to establish an agreed format among journalists (Thompson, 2010). In order to establish its public diplomacy style, the management of TRT World constructed a style guide mimicking that of other media outlets such as Al Jazeera and BBC. Participant#14 described TRT World’s style guide as ‘plagiarised’ from Al Jazeera’s international guide. The style guide, which we were able to obtain a copy of, is oriented towards Turkey’s position on events. For instance, DHKP (Revolutionary People’s Liberation Party-Front) and PKK (Kurdistan Workers’ Party) would be described as terrorist organisations. The guide instructed journalists to refer to the Syrian government as a ‘regime’ for being an un-democratically elected government. Our respondents told us that they were asked to refer to the Turkish Islamic Gülen Movement (GM), which was blamed for leading the failed 2016 coup attempt in Turkey, as a terrorist group.

In addition to that, TRT World established a research centre to produce reports and organise conferences. From the interview data, it was argued that this centre was established to follow the same steps as the Al Jazeera Centre for Studies. One London based journalist sees this centre as a soft power tool created by Turkey to reach out to academics and influencers. According to Participant#5, ‘they would invite opinion leaders to Istanbul, accommodate them in nice hotels and give them free food, so they will start thinking nicely of Turkey’.

### The digital department

Early on in its development, the management of TRT World reached out to online audiences by digitising its content. The digital department was outsourced to another company, a step that our respondents considered ‘unique’ as a newsroom strategy. TRT World management invested heavily in this digital department and supplied the organisation with staff and equipment to separate it from the TV section, providing it with its own studio and cameras to produce outputs attractive to online audiences.

Our respondents noted that the TV and the digital department, for the most part, worked separately and did not feed into each other. One of our respondents commented that they believed that TRT management wanted to establish a similar digital model to the Al Jazeera-run online platform AJ+. Another respondent, Participant#13, observed how the systems were redundant:

They would do some separate pieces like ‘*5 things to know about the war in Iraq.*’ They had short videos rounding up the news. They have a newscast, a green screen, and a presenter (Participant#13, 2018).

The digital department was made responsible for TRT World’s social media accounts and the website. The channel has more than 1 million subscribers on YouTube, with about 393 million views to the date of writing.

The majority of our respondents pointed out that the content produced by the digital department was more biased and one-sided. One example was recounted by participant#10, a former Istanbul-based journalist:

They made a very strong video against El-Sisi [the Egyptian President]. For instance, while we in TV would report about it with more balance. . .They [the digital department] made many videos that support the Muslim Brotherhood that is not reflected on TV (Participant#10, 2018).

Participant#12 described the digital department’s coverage as ‘more biased, and more shameless’ compared with the TV department. Participant#15 expressed the belief that this slanted output was because this digital department did not have to abide by any broadcast regulations. Three of the respondents said that they complained about the bias in the content of the videos produced by the digital department.

### A party broadcaster?

TRT World is the newest addition to Turkey’s national broadcaster TRT. TRT, as a network, is considered the main national Public Service Broadcaster (PSB) in Turkey. The organisational structure of TRT World – including its autonomy from the state – is controversial. Even though TRT World positions itself as an independent organisation that is not controlled by the state, many observers disputed this claim. In 2019, the US Department of Justice ordered TRT Cooperation’s office in the US – that mainly produces content for TRT World – to register as a foreign agent for being a ‘publicity agent and information-service employee in the US for the Government of Turkey’ (The US Department of Justice, 2019). The Department of Justice justified its resolution by stating that the Turkish government controls TRT Corporation by deciding the size of grants allocated each year and the amount of tax revenues the network can receive (The US Department of Justice, 2019). It is reported that about 70 percent of TRT’s funds come from taxes, and the rest comes from government grants, and advertising revenues (McPhail and Phipps, 2019).

Despite this contention, the majority of our respondents asserted that TRT World is a public broadcast channel that does not rely on the government fund. Repeatedly and across most of the interviews, respondents compared the channel’s funding model with other public service broadcasters such as the BBC’s. Our respondents argued that TRT World is a PSB on the basis that there is a 2 percent tax on household electricity bills to support TRT Corporation. However, Participant#7, a former Istanbul-based TRT World journalist, argued that TRT World only advertised itself as a PSB to ‘gain legitimacy’:

On paper, [being a PSB] might sound good to them, but in reality, it is not. If they were a public broadcaster, it would have given the same time in the election reports to the ruling party and to the opposition parties. They never bring the opposition to comment (Participant#7, 2018).

Although the majority of respondents referred to TRT World as a PSB due to its being mainly funded through a non-voluntary tax, the majority also expressed its bias towards Erdoğan’s AKP and implied that it does not prioritise public service. Participant#13 believed that TRT World is a government-backed broadcaster due to the constraints imposed on the editorial policies. Another respondent offered a similar observation about how the government possessed a far greater ability to reduce budgets, make cutbacks and reward certain individuals than it should have if the channel were a PSB.

Although the initial plan was to use TRT World as an impartial and professional public diplomacy tool, this could not be sustained. Throughout the interviews, participants argue that the TRT World’s newsroom was not editorially free. The control began with a policy of hiring senior managers who had close ties to Erdoğan and the AKP. It was pointed out that senior people at TRT would have not only been chosen by the government but were also actively affiliated with the government. One of the respondents noted that professional Turkish journalists were discouraged so they could leave, creating a vacancy for loyal people from the AKP. Participant#13 commented:

In the management, the higher up you go, it was apparent that you had to be from the loyalists of the Party . . . they were clearly in direct contact with Erdoğan’s people and were pushing for that Party (Participant#13, 2018).

The affiliation with the government did not end with the hiring of senior managers. In the interviews, participants viewed that the TRT Corporation was run and overseen by the Ministry of Interior. An interviewee remarked that the Chairman of TRT also acted as Erdoğan’s media advisor.

However, the day-to-day activities on the channel were not closely monitored by the government. Participant#7, a former Istanbul-based TRT World journalist, argued that appointing AKP loyalists in managerial positions made frequent direct governmental intervention less necessary. As discussed above, interviews suggested that journalists are not explicitly directed as to which stories to report on; however, there were occasional reports of censorship being exercised. As Participant#12, a former Istanbul-based TRT World journalist, recalled, their producer would sometimes receive ‘a call’, and a story would be canceled. Other respondents reported that there were several stories related to domestic Turkish politics that were never approved. From our interviews, it was noted that stories related to President Erdoğan had to be pre-approved by the President’s office before they could be written and broadcasted. One of the respondents commented that they were required to represent the President as if he were ‘the nicest person on earth’.

**Figure 1.**
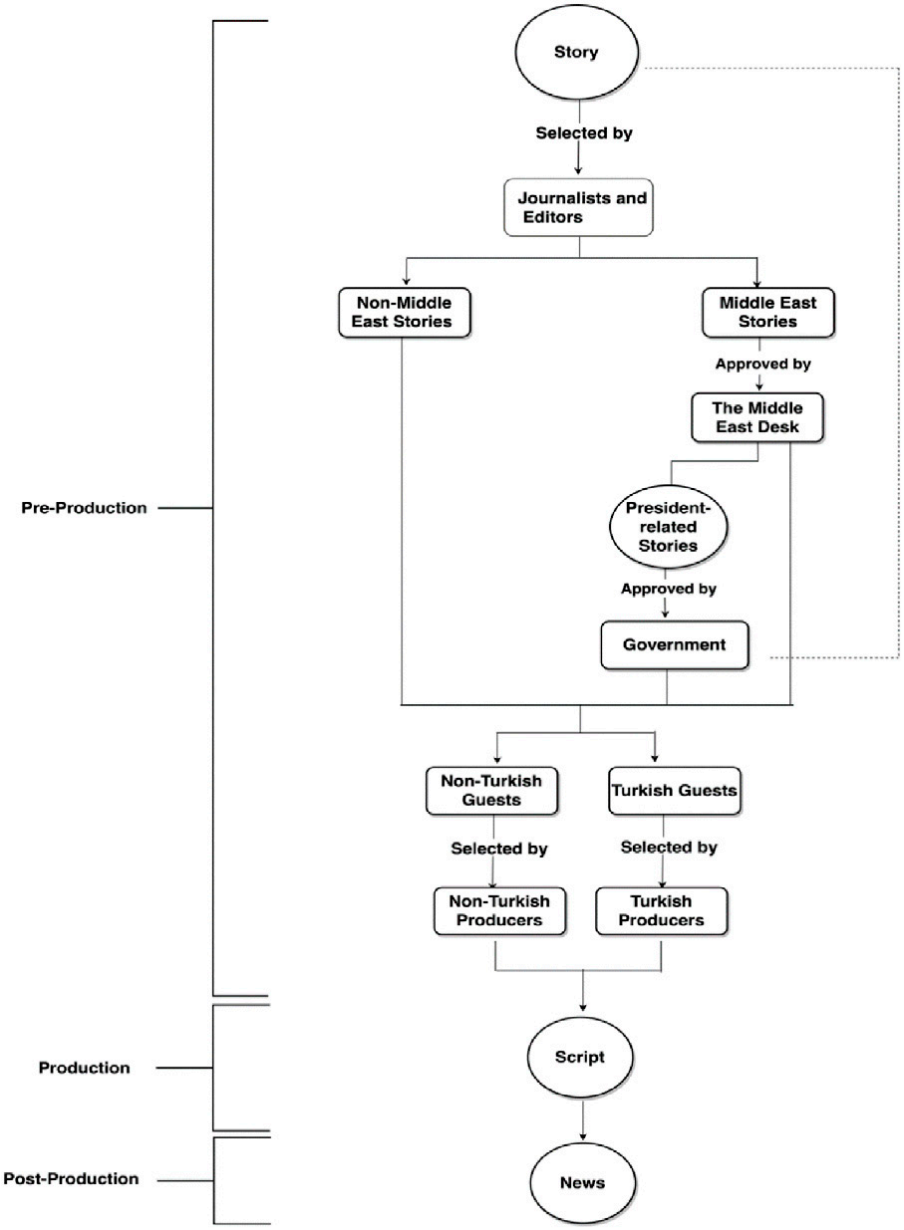
News production workflow within TRT World. *Source*: Based on our interviews with TRT World journalists collected from January 2018 to March 2019. *Note*: A circle represents the story development, boxes are the decision-makers at the channel, thin arrows represent the sequence which the story follows. Dotted lines refer to the new process of direct intervention from the government in the channel’s output following the 2016 failed coup attempt.

In general, censorship was sometimes imposed on the Middle East-related stories (See Figure 1). These stories could be on Turkey or on neighbouring countries. The experience of this censorship varied among the respondents. Participant#9, an Istanbul news producer, commented that they were able to cover all stories in Turkey, including topics related to human rights violations. However, participant#2, an Istanbul-based senior journalist, stated that they would be fired if they undermined the Turkish government at TRT World.

Much of the control came from the Middle East news desk, which was responsible for covering stories on Turkey and the Middle East. Participant#10, who was based in Istanbul, described the Middle East desk as ‘the censorship desk’, referring to their process of selecting news stories. The responsibilities of the desk included translating sources, facilitating access to correspondents and providing clarifications for non-Turks. This desk was the first point where a certain Middle East-related story would be approved or ignored. Participant#12 elaborated:

Most of the Middle East news desk people were pretty good. They did know what they were talking about. I guess there was a time you would want to report something that would reflect badly on the Turkish government, and they would say we have to get our response first from the Turkish government (Participant#12, 2018).

Notably, the opposition was rarely represented on the channel. All the respondents mentioned that TRT World did not provide enough space to the opposition political parties in Turkey while covering domestic news. The channel has failed in constructing a sense of legitimacy and trust in their relationship with those opposed to Erdoğan’s government. On the occasions they would be invited to participate in any of the shows, representatives of the opposition parties would refuse to be interviewed by TRT World. One of the respondents observed that the Turkish opposition has always criticised the TRT Corporation, in general, for not giving them equal access to coverage and airtime.

Opposition or not, Turkish guests were hand-selected by a Turkish employee at TRT World. Although Participant#11, a London-based journalist, seemed to have a very positive experience at TRT World, they said that the only thing they were not able to do freely was to select Turkish guests when needed. They had to let the Istanbul office select guests instead. Such censorship did not occur when selecting international non-Turkish guests. Participant#13 lamented:

For international guests, it will be the international guest booker and we had quite good expats doing this job. For Turkish guests, we would get a Turkish filter of who did they want to bring on (Participant#13, 2018).

The above examples show that TRT World’s ambitions to be perceived as an autonomous and impartial international channel could not succeed. The management’s interest in sustaining the channel’s image of independence diminished over the years and it became clear that the channel does not hide its party loyalty and serves Erdoğan’s national and international agendas. The next section thus discusses the organisational model of the channel that hindered asserting its initial public diplomacy goal.

### The Turkification of TRT World

‘Turkification’ is a term used to describe a central strategy associated with the Turkish nation-building project, a strategy of creating a dominant and homogenised Turkish identity (Demir, 2017). A similar process was undertaken at TRT World to ‘Turkify’ the organisation. From the interviews, respondents indicated that the majority of people employed at TRT World were – and continue to be Turkish. All respondents agreed that all top managers in the channel across the different bureaus are Turkish citizens, though this was not the case when the channel started in 2015. According to our interviews, when the channel started, there were no Turkish managers apart from the chairman and the vice-chairman. Gradually, the people in the top positions were replaced by Turks. Participant#7 noted that some of these new Turkish managers had no journalism experience prior to joining TRT World.

This process did not only affect the managers. Turkification spread to include all journalists at the channel, with the strategy embedded in the hiring scheme. They hired a large number of Turkish interns to learn from and observe the professional international journalists appointed at the first hiring wave. One of the journalists who witnessed the establishment of the channel explained that the TRT management had planned from the outset to replace the expats with Turks to work for the channel. Participant#12 noted:

I always felt that our positions there were temporary. . . the idea [was] that further down the line, this [TRT World] would be a Western-oriented English-language 24-hour-global news channel entirely run by Turkish people (Participant#12, 2018).

Currently, the channel is a ‘Turkish-run’ operation. Participant#14, who has worked for the channel for years, told us that at the beginning, the senior editors were non-Turks. Over the years, this changed and TRT World is currently run by Turks who have political agendas.

The Turkification process has successfully promoted the perception that working for TRT World is a national duty. Being part of TRT World came to represent a shared sense of patriotism and nationalism. Some of the respondents observed that Turkish journalists working at TRT World expressed a sense of pride for serving their nation. Participant#10 talked about their colleague who described the work at TRT World as a national service that was more important than enrolling in the army.

Participant#5, a London-based journalist, noted that the Turkification process was always a top priority, helping to ensure that the decisions were not made by ‘some foreigners’ and that the Turkish perspective would be projected accurately. One of the London-based respondents put the ratio between foreigners and Turks within TRT World at 1:3.

### The post-2016 coup attempt era

TRT World’s public diplomacy ambition was greatly impacted by Turkey’s domestic politics. Turkey shifted its public diplomacy to more of an authoritarian approach (Uysal and Schroeder, 2019). The failed coup attempt in 2016 in Turkey reflects how repressing media freedoms in a country eventually impacts its international broadcaster. Once the coup failed, Erdoğan tightened his grip over media in the country to curb the circulation of information that might threaten his regime. Erdoğan used this abortive coup to justify a sweeping purge of political opponents from media institutions. Since the coup, more than 170 outlets have been shut down in addition to the arrests of dozens of journalists (Waldman and Caliskan, 2019). This policy continues and has involved sustained pressure on traditional and digital media and placed Turkey in a state of emergency for more than 2 years. Like all other media institutions, TRT World was affected by this purge.

Many TRT World journalists who were not clearly loyalists were fired. One of the respondents stated that the firing wave included journalists who studied in Gülen schools, those who downloaded the encrypted mobile application ‘ByLock’, which was used by the Gülen movement, and others who were allegedly Gülen supporters. Participant#10 recounted:

There have been some colleagues inside TRT World who have been temporarily dismissed and then reintegrated because of being, allegedly, with the Gülen organization, which later had been proved wrong (Participant#10, 2018).

According to our interviews, the coup had a significant influence on the editorial policy of the channel. At the birth of TRT World, the management intended to westernise the channel, followed by gentle ‘Turkification’. In the aftermath of the coup attempt, the agenda changed to mainly focus on Turkey-related stories. Participant#13 observed:

The whole reason for the geneses of the project [TRT World] was to be more Western. This has changed for a number of reasons. First of all, the political development in Turkey; there was an attempted coup in Turkey, which has changed everything politically. There is an authoritarian track in the government that was not there before (Participant#13, 2018).

The impact has extended to content creation. Our respondents observed that the channel was later used to promote Erdoğan and to denigrate the Gülen movement. For weeks, promotional videos supporting Erdoğan were aired on TRT World as part of the newscast. Respondents who witnessed the aftermath of the coup expressed the concerns they had about these videos. The promotional videos were forcibly added to the TRT World’s agenda without the consent of the producers. Participant#12 elaborated:

Promotional videos, some were 20 to 30 seconds long, they would run in the back of a news program or during the break of a news program. They were made to look like a part of the news . . . some sort of promotional video about the coup and describing the assaulted democracy and how the Gülenists are behind it and how America refused to exodus Gülen and all of that. You start asking in the newsroom, did you make that? Nobody had any involvement in it, and somebody else signed off and slotted it in the TV schedule independently from the newsroom (Participant#12, 2018).

These videos flooded TRT World’s website and social media pages. The online promotional videos were also about how the Gülenists tried to take over Turkey and how they stood up for democracy.

The coup attempt had further stimulated the nationalist sense of the Turkish staff. One of the respondents noted that some of the Turkish journalists pushed a nationalist ideology and promoted a pro-AKP agenda following the attempted coup. Turkish journalists became more firmly supportive of Erdoğan’s government than ever before. Thus, the AKP’s agenda became more embedded in the channel and hindered the practice of impartial and professional journalism.

## Conclusion

This paper is relevant to the contemporary discussion about the role of international broadcasting in public diplomacy. It provides a model of an international broadcaster that originates in a country where press freedom is restricted. While this paper does not study the effect of TRT World on foreign policy, it attempts to analyse the editorial dynamics of TRT World and the factors that have impacted the style of the channel as a public diplomacy tool.

After interviewing 16 TRT World journalists and having examined the style guide of the channel, we can conclude that the limited media freedom in Turkey has impacted how TRT World operates. Although the channel’s team included professional journalists aspiring to initiate a credible public diplomacy tool for Turkey in 2015, the channel could not separate itself from the censored media ecosystem in Turkey for too long.

In international broadcasting, credibility matters. Without it, public diplomacy cannot function to transmit soft power resources effectively (Nye, 2008). One of the factors that contribute to the success of international broadcasters is their ability to tune in to the expectations and information needs of foreign audiences (Youmans and Powers, 2012). An impression of direct government interference in the editorial process of an international broadcaster undermines its public diplomacy role (Cull, 2008). TRT World aimed to build this trust at first by positioning itself as a public service broadcaster, building a research centre, establishing a resourceful digital department and hiring world-renowned journalists. In the early days of the channel, it functioned as a public diplomacy project to translate Turkey’s soft power resources to the world and enhance its image.

However, the autonomy of TRT World’s project was fragile. TRT World’s ambition to implement a public diplomacy style could not survive the Turkish censorship and repression that started to creep into the channel over the years. This was mostly prominent following the attempted coup of 2016 when media restrictions in Turkey increased and impacted TRT World’s editorial process and structure. This interference in the channel can be seen through the Turkification process, the exclusive hiring of AKP loyalists in managerial positions, the forcible promotion of Erdoğan’s AKP loyalists following the coup attempt and the channel’s censorship of presidential and regional stories.

By studying the TRT World case, we argue that the local media ecosystem and laws in a country determine the style of its international broadcasting. Under authoritarian regimes, this could mean turning the well-funded and ambitious international broadcaster into another government mouthpiece that fails to establish credibility abroad. Although repressive regimes aspire to build strong public diplomacy tools to represent them as democratic and fair states, they continue to impose further restrictions on their local media to protect their power. The domestic media ecosystem and the limited censorship in these regimes eventually impact their international broadcasting style.

In order to build a successful public diplomacy tool, these regimes need to be ready to accept political challenges and criticism. If a government does not articulate the values of democracy and freedom in their own countries, efforts of public diplomacy, such as international broadcasting, will be ineffective. The embedded media censorship and government interference are the two main reasons international broadcasting backed by authoritarian states could, at some point, turn from a public diplomacy tool into a party mouthpiece.
